# Daytime sleepiness and emotional and behavioral disturbances in Prader-Willi syndrome

**DOI:** 10.1007/s00431-022-04439-2

**Published:** 2022-03-22

**Authors:** Catherine S. Choong, Gillian M. Nixon, A. Marie Blackmore, Wai Chen, Peter Jacoby, Helen Leonard, Antony R. Lafferty, Geoff Ambler, Nitin Kapur, Philip B. Bergman, Cara Schofield, Chris Seton, Andrew Tai, Elaine Tham, Komal Vora, Patricia Crock, Charles Verge, Yassmin Musthaffa, Greg Blecher, Andrew Wilson, Jenny Downs

**Affiliations:** 1 Telethon Kids Institute, 15 Hospital Avenue, Nedlands, Western Australia 6009 Australia; 2 Perth Children’s Hospital, Nedlands, Western Australia Australia; 3 Sophia Children’s Hospital, Erasmus University Medical Centre, Rotterdam, Netherlands; 4 Monash Children’s Hospital, Clayton, Victoria Australia; 5 Monash University, Clayton, Victoria Australia; 6 Child and Adolescent Health Service, Perth, Western Australia Australia; 7 Fiona Stanley Hospital, Murdoch, Western Australia Australia; 8 Curtin University, Bentley, Western Australia Australia; 9 Canberra Hospital, Garran, Capital Territory Australia; 10 ANU Medical School, Canberra, Capital Territory Australia; 11 The Sydney Children’s Hospitals Network, Westmead, New South Wales Australia; 12 The University of Sydney, Sydney CBD, New South Wales Australia; 13 Queensland Children’s Hospital, South Brisbane, Queensland Australia; 14 The University of Queensland, Brisbane, Queensland Australia; 15 La Trobe University, Bundoora, Victoria Australia; 16 The Children’s Hospital at Westmead, Westmead, New South Wales Australia; 17 Woolcock Institute of Medical Research, Glebe, New South Wales Australia; 18 Women’s and Children’s Hospital, North Adelaide, South Australia Australia; 19 John Hunter Children’s Hospital, Newcastle, New South Wales Australia; 20 The University of Newcastle, Newcastle, New South Wales Australia; 21 Hunter Medical Research Institute, Newcastle, NSW Australia; 22 The University of New South Wales, Kennington, New South Wales Australia; 23 Queensland Children’s Hospital, South Brisbane, Queensland Australia; 24 Logan Hospital, Meadowbrook, Queensland Australia; 25 The Sydney Children’s Hospitals Network, Randwick, New South Wales Australia; 26 The University of Western Australia, Nedlands, Western Australia Australia

**Keywords:** Intellectual disability, Genetic disorder, Mental health, Sleepiness

## Abstract

Individuals with Prader-Willi syndrome (PWS) often have excessive daytime sleepiness and emotional/behavioral disturbances. The objective of this study was to examine whether daytime sleepiness was associated with these emotional/behavioral problems, independent of nighttime sleep-disordered breathing, or the duration of sleep. Caregivers of individuals with PWS (aged 3 to 25 years) completed the Pediatric Sleep Questionnaire (PSQ), Epworth Sleepiness Scale for Children and Adolescents (ESS-CHAD), and the parent version of the Developmental Behavior Checklist (DBC-P). Sleep adequacy was adjusted for age by computing sleep duration against age-specific recommendations. The associations between ESS-CHAD and the total DBC and its subscale scores were evaluated by linear regression, adjusted for sleep-related breathing difficulties, sleep adequacy, and body mass index (BMI). There were 54 responses for individuals with PWS (including 22 males) aged 4.4–24.0 (mean 12.5) years. Daytime sleepiness predicted a substantial proportion of the variance in total DBC-P scores in the unadjusted model (28%; *β* = 0.028; *p* < 0.001) and when adjusted for sleep adequacy, BMI, and sleep-related breathing difficulties (29%; *β* = 0.023; *p* = 0.007). This relationship was not moderated by BMI *Z*-scores, but the relationship was more prominent for children younger than 12 years than for children older than 12 years.

*Conclusions*: These findings provide preliminary novel evidence that daytime sleepiness may drive the expression of emotional/behavioral disturbances, and should be explored as a potential modifiable risk factor for these disturbances in PWS, particularly pre-adolescent children.

**What is Known:**

*• Individuals with Prader-Willi syndrome (PWS) commonly experience excessive daytime sleepiness and exhibit emotional/behavioral disturbances.*

*• In the typically developing population, sleepiness is associated with emotional/behavioral disturbances, independently of sleep-disordered breathing.*.

**What is New:**

*• This study found evidence for a direct link between daytime sleepiness and emotional/behavioral disturbances, independent of sleep-related breathing difficulties, sleep adequacy, and body mass index.*

*• Excessive daytime sleepiness may be a modifiable risk factor for emotional/behavioral disturbances in PWS.*

## Introduction

Prader-Willi syndrome (PWS) is a neurogenetic condition due to defects at q11–13 on chromosome 15, occurring in approximately 1 in 16,000 live births [1]. It affects both males and females, and is characterized by a unique behavioral profile, associated with developmental delay and intellectual disability. In infancy, hypotonia and poor sucking lead to feeding difficulties, but during childhood, an excessive appetite for food develops (hyperphagia) [2], leading to obesity and obesity-related health problems. Other associated problems include sleep disturbances and emotional/behavioral problems [3, 4].

Nighttime sleep-disordered breathing, particularly obstructive sleep apnea (OSA), is common in PWS: a meta-analysis of children and adolescents with PWS reported a prevalence of 80%, including 25% with severe OSA [5]. Excessive daytime sleepiness is also common. One study reported that half of individuals with PWS experience sleepiness (52%) and the majority have regular daytime naps (89%) [6]. Symptoms of narcolepsy have been reported in 36% of children with PWS [5]. Nighttime OSA is not the only cause of daytime sleepiness in children with PWS as excessive daytime sleepiness can occur without OSA and often persists after successful treatment for OSA. Therefore, daytime sleepiness has been considered primary (rather than as secondary to nighttime sleep problems), and postulated to be related to hypothalamic dysfunction [3].

Symptoms of emotional/behavioral disturbance above clinical thresholds are more common in children with PWS (74%) than in children with other genetic disorders associated with intellectual disability (Down syndrome [32%], 22q11.2 deletion syndrome [46%], fragile X syndrome [61%], Williams syndrome [67%]) [7]. The most common emotional/behavioral problems in PWS include temper tantrums, obsessions, distress over small changes, repetitive questioning, and being abusive [8, 9]. So far, in the literature, these challenging problems have been assumed to be the clinical expression of the unique clinical phenotype of PWS. An alternative explanation may be that some of these emotional/behavioral problems are secondary to daytime sleepiness. If so, this would offer new insights for alternative treatment strategies.

In the typically developing population, sleepiness is associated with behavior problems independently of sleep-disordered breathing [10, 11] Studies on nighttime sleep-disordered breathing and emotional/behavioral disturbances in PWS have yielded mixed results, including positive associations [12], no associations [13, 14], equivocal findings [15, 16], and even a negative association [17]. On the other hand, there is some evidence of associations between daytime sleepiness and emotional/behavioral problems measured [16, 18]. The roles of other potential influencing factors (sleep-related breathing difficulties, sleep adequacy, and body mass index [BMI]) on the relationship between daytime sleepiness and behavior have also not been investigated.

This study aimed to investigate the associations between daytime sleepiness and emotional/behavioral disturbances in a community sample of individuals with PWS independently of sleep-related factors (sleep-related breathing difficulties, sleep adequacy, and BMI). A secondary aim was to determine the extent to which any observed associations between daytime sleepiness and emotional/behavioral disturbances may differ between younger and older age groups and between individuals who are overweight and not overweight.

## Methods

### Participants

The data were from the Australasian Prader-Willi Syndrome Registry. This Registry was set up by investigators at Telethon Kids Institute and clinicians of children and adolescent with PWS in tertiary hospitals for children across Australia. Families of young people with PWS could enroll in the registry directly or be referred by their clinician. Participants were eligible for inclusion in this study if they were a caregiver of an individual with PWS who was aged 25 years or younger. Data were collected between May 2018 and March 2021.

This research was approved by the Western Australian Child and Adolescent Health Service Human Research Ethics Committee (RGS1112) at Perth Children’s Hospital under the National Mutual Agreement, with local governance approval from all participating sites (Monash Health RES-18–0000-774X; Women’s and Children’s Health Network HREC/18/OTHER/094, SSA/18/WCHN/184; Queensland Children’s Hospital SSA/19/QCHQ/4919; La Trobe University WACAHealthServiceRGS1112; Royal Children’s Hospital 2019.096; Sydney Children’s Hospital Randwick 2019/STE04456; Westmead Children’s Hospital 2019/STE04455; John Hunter Children’s Hospital 2019/STE04457; Canberra Children’s Hospital 2019/STE/00099). All participants gave informed consent.

### Measures

Data were extracted from a survey of health, functioning, and quality of life in individuals with PWS and their families. Data pertaining to sleep, emotional/behavioral problems, and demographic characteristics are reported here.

The sleep-related questions were selected to describe daytime sleepiness, adequacy of nighttime sleep, breathing problems during sleep, and factors that might impinge on these. These included questions about duration of sleep (“how many hours of sleep does your child get most nights?” coded as 9–11 h, 8–9 h, 7–8 h, 5–7 h, and less than 5 h); previous or current diagnoses of OSA, central sleep apnea (CSA), or narcolepsy; history of ear, nose, and throat surgeries; current breathing support during sleep (supplemental oxygen, continuous positive airway pressure, and bi-level positive airway pressure); the Pediatric Sleep Questionnaire (PSQ) [19]; and the Epworth Sleepiness Scale for Children and Adolescents (ESS-CHAD). 20]. The PSQ lists 22 sleep behaviors, each rated as either present or absent. A score of ≥ 0.33 of a total score of 1 is considered predictive of a diagnosis of OSA [19]. The PSQ subscale of 9 items that loaded on a factor entitled “breathing” [19] was used in the current analysis. The ESS-CHAD is an 8-item scale assessing daytime sleepiness [20]. All items (rated on a 0–3 scale) are summed to yield a total score out of 24, which were classified as five levels with scores between 11–12, 13–15, and 16–24 described as mild, moderate, and severe excessive daytime sleepiness, respectively.

Emotional/behavioral difficulties were assessed using the parent-completed form of the Developmental Behavior Checklist (DBC-P) [21], a 96-item questionnaire designed to assess emotional/behavioral problems in individuals with an intellectual disability. Each item is rated as 0 (“not true”), 1 (“somewhat or sometimes true”), or 2 (“very true or often true”). The items can then be averaged to yield a total DBC score and subscale scores. The subscales used in this study were *disruptive/antisocial*, *self-absorbed*, *communication disturbance*, *anxiety, social relating*, *depression*, and *autistic-relating*. Parent-completed versions of the DBC and ESS designed for individuals up to age 18 were applied to young adults in this study to allow comparison of results across the age range.

Demographic variables included age and sex, body weight and height to give BMI, other diagnoses, and current and past use of growth hormone. Laboratory results of genetic subtype were collected, with participants’ permission, directly from their responsible clinicians.

The survey was administered online, on paper, or by phone interview. The REDCap (Research Electronic Data Capture) platform was used to create the survey and collect responses.

### Data management

BMI was converted to an age-standardized modified *Z*-score using the Centers for Disease Control and Prevention growth charts [22], and then converted to a binary variable with a cut-point of *Z* = 2.0 [23]. From number of hours of sleep per night, a binary *sleep adequacy* variable was derived, based on the National Sleep Foundation’s age-dependent recommendations, with adequacy of sleep defined as a reported duration at or above the recommended amount for age [24]. To examine the effects of age as a moderating variable in the association between daytime sleepiness and emotional/behavioral disturbances, individuals were stratified into those younger than 12 years (*n* = 26) and those 12 years and older (*n* = 28). Mean subscale scores and total scores were computed for the PSQ and DBC based on non-missing values. The ESS-CHAD total score was computed for all participants with complete ESS-CHAD data (*n* = 51), and descriptive statistics were computed for unimputed data. For the regression analyses, the only three missing data points on the ESS-CHAD (all on item 8) were replaced with the sample mean for that item in order to obtain a total ESS-CHAD for all participants. Total and subscale DBC-P scores were reported as mean item scores on a scale of 0 to 2 for easier comparison of subscale scores.

### Data analysis

Data analyses were conducted using STATA/SE 16.1 for Windows (Revision 21 Jan 2021). The chi-squared test of independence was used to evaluate the association between PSQ (categorized as above or below 0.33) and ESS-CHAD. Univariate linear regression was conducted to evaluate the associations between the ESS-CHAD score and the scores of total DBC and its subscales. Multivariate regression was conducted adjusting for the potential confounding effects of sleep adequacy, BMI age-standardized *Z*-scores, and sleep-related breathing difficulties (the 9-item breathing difficulties subscale from the PSQ). To account for the multiple dependent variables, global *p* values were computed in both the unadjusted and adjusted models using multivariate Wald tests [25]. To explore the moderating effect of age and BMI on this association, these two variables were dichotomized and included in the adjusted regression model as represented by an “ESS-CHAD by age” interaction term and an “ESS-CHAD by BMI” interaction term. Alpha was set at 0.05. Two analyses of covariance (ANCOVAs) were performed as post hoc analyses comparing the levels of breathing difficulties and daytime sleepiness of those on growth hormone and those not, with age as the covariate.

## Results

### Participant characteristics

Surveys were completed by caregivers of 54 individuals with PWS aged 4 to 24 years (Table 1). Almost half (48%) had a BMI age-standardized *Z*-score over 1.0, 35% over 2.0, and 20% over 3.0. Three quarters (74%) were taking growth hormone at the time of the survey.

**Table 1:** Characteristics of individuals with PWS in the study (*n* = 54)

Sleep variables	*M* (*SD*) range	*n*	%
Age	12.5 (5.4) 4.4–24.0		
Males to females		22:32	40:60
Genetic subtype			
Paternal deletion		22	41
mUPD		19	35
Other		7	13
Unknown		6	11
Body mass index–modified *Z*-score	1.71 (2.19) −0.53–10.31		
Growth hormone: currently (*n* = 50)		37	74
Growth hormone: ever (*n* = 54)		50	93
*Diagnosis or treatment*			
Ever diagnosed with obstructive sleep apnea		26	48
Still has obstructive sleep apnea		8	15
Receives treatment for obstructive sleep apnea		4	7
Ever diagnosed with central sleep apnea		8	15
Still has central sleep apnea		6	11
Receives treatment for central sleep apnea		2	4
Ever diagnosed with narcolepsy		3	6
Still has narcolepsy		3	6
Receives treatment for narcolepsy		3	6
Ear, nose, and throat surgery (including adenoidectomy, tonsillectomy, adenotonsillectomy, ventilation tube placement)		25	46
Other ear, nose, and throat surgery		7	13
Support with breathing during sleep		6	11
Supplemental oxygen		6	11
Positive pressure support		5	9
*Individuals achieving recommended hours of sleep per night* (*n* = 54)			
Preschoolers (3–5 years): ≥ 9 h sleep/night (*n* = 6)		5	83
School-age children (6–13 years): ≥ 9 h sleep/night (*n* = 26)		18	69
Teenagers (14–17 years): ≥ 7 h sleep/night (*n* = 10)		9	90
Young adults (18–25 years): ≥ 7 h sleep/night (*n* = 12)		11	92
*Total Pediatric Sleep Questionnaire* (*n* = 54)			
Total Pediatric Sleep Questionnaire	0.41 (0.19) 0.45–0.85		
Total Pediatric Sleep Questionnaire ≥ 0.33 (suggesting obstructive sleep apnea) [19]		37	69
Pediatric Sleep Questionnaire Breathing subscale	0.28 (0.27) 0–1		
*Epworth Sleepiness Scale* (*n* = 54)			
Total Epworth Sleepiness Scale	9.50 (4.79) 0.0–24.0		
0–5 lower normal daytime sleepiness		8	16
6–10 higher normal daytime sleepiness		26	51
11–12 mild excessive daytime sleepiness		6	12
13–15 moderate excessive daytime sleepiness		6	12
16–24 severe excessive daytime sleepiness		5	10

Pediatric Sleep Questionnaire (possible range 0–1); higher score means more sleep difficulties

Epworth Sleepiness Scale (possible range 0–24); higher score means more sleepiness

### Sleep variables

In this study, 43/54 (80%) participants met the National Sleep Foundation’s age-dependent recommendations for adequate hours of sleep per night. Approximately half (46%) of the young people with PWS had undergone ear, nose, and throat surgery, and half (52%) had previously been diagnosed with either OSA (48%), CSA (15%), or narcolepsy (6%), but only a quarter of these (24%) still had any of those diagnoses at the time of this survey, and 11% were actively receiving treatment for OSA, CSA, or narcolepsy. However, 69% (37/54) had a total PSQ score ≥ 0.33, suggesting that OSA symptoms were still present. A total PSQ ≥ 0.33 was associated with excessive daytime sleepiness (*p* = 0.004): 46% (17/37) of individuals with a high total PSQ score had excessive daytime sleepiness, whereas only 6% (1/17) of those with a low total PSQ score had excessive daytime sleepiness.

### Emotional/behavioral outcome

The mean (*SD*) for the total DBC-P was 0.53 (0.25), for the disruptive/antisocial subscale was 0.52 (0.27), for the self-absorbed subscale was 0.52 (0.26), for the communication disturbance subscale was 0.49 (0.25), for the anxiety subscale was 0.56 (0.32), for the social relating subscale was 0.54 (0.28), for the depression subscale was 0.49 (0.29), and for the autism screening algorithm was 0.52 (0.25).

### Associations between sleep and emotional/behavioral problems

Associations between daytime sleepiness and emotional/emotional/behavioral disturbances are presented in Table 2. Daytime sleepiness was associated with a substantial proportion of the variance in total DBC-P scores in the unadjusted (28%) and adjusted (29%) models. In the adjusted model, a one-point increase in the ESS-CHAD total score corresponded to an increase in total DBC (on a scale that could range from 0 to 2) of 0.023 (95% CI: 0.007 to 0.040) equating to 4.5 points on the 196-point DBC scale. Daytime sleepiness predicted variance in all the DBC subscales except for the communication disturbance subscale in the adjusted model (Table 2).

**Table 2:** Regression for DBC-P total and subscales predicted by ESS total (*n* = 54)

DBC-P outcome	Unadjusted model^a^			Adjusted model^bc^		
	Coefficient (95% CI)^d^	*p* value	*R* square	Coefficient (95% CI)^d^	*p* value	*R* square
Total	.028 (.015–.040)	< .001	.28	.023 (.007–.040)	.007	.29
Disruptive/antisocial	.028 (.014–.041)	.001	.25	.020 (.002–.038)	.028	.28
Self-absorbed	.026 (.013–.040)	< .001	.23	.027 (.009–.045)	.004	.23
Communication disturbance	.017 (.003–031)	.015	.11	.009 (− .004–.033)	.127	.13
Anxiety	.029 (.013–.046)	.001	.19	.011 (.001–.046)	.041	.21
Social-relating	.028 (.014–.043)	< .001	.23	.010 (.006–.045)	.013	.24
Depression	.035 (.022–.049)	< .001	.35	.019 (.004–038)	.019	.44
Autism	.025 (.012–.038)	< .001	.22	.009 (.009–.044)	.005	.22

^a^Unadjusted test for global *H*_0_ that ESS prediction of all subscales was nil: *p* = .0002

^b^Adjusted for sleep adequacy, BMI-modified Z-score, and PSQ Breathing subscale

^c^Adjusted test for global *H*_0_ that ESS prediction of all subscales was nil: *p* = 0.0714

^d^Coefficient is the modeled increase in DBC-P score per unit change in ESS

**Fig. 1:**
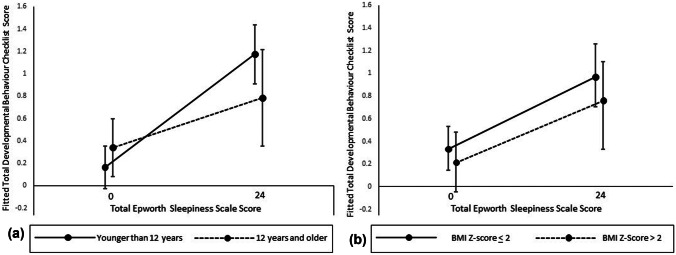
Regression lines for sleepiness against total Developmental Behavior Checklist scores for **a** young and older individuals and **b** individuals with lower and higher body mass index (BMI) *Z-*scores

Figure 1 shows that the regression coefficient for association between daytime sleepiness and total DBC scores in pre-adolescent children (0.042) was twice that of the adolescents and young adults (0.019), suggesting that sleepiness is more strongly associated with emotional/behavioral problems in children than in adolescents and young adults (*p* = 0.115). BMI, on the other hand, did not affect the regression coefficients (0.026 for those with BMI *Z*-score < 2; 0.023 for those with BMI Z-score > 2) and the interaction term for BMI by ESS-CHAD was not statistically significant (*p* = 0.788).

### Associations between current use of growth hormone and sleep factors

Two post hoc analyses were performed to determine whether those currently on growth hormone differed from those not on growth hormone regarding daytime sleepiness and breathing difficulties, adjusting for age. There was little difference in sleep-related breathing difficulties between those who were currently on growth hormone (*M* = 0.21; *SD* = 0.21; *n* = 37) and those who were not (*M* = 0.42; *SD* = 0.30; *n* = 13), with a small between-group difference (*β* = −0.148 (95% CI: −0.356, 0.059), *p* = 0.16). Nor was there any significant difference in daytime sleepiness between those on growth hormone (*M* = 8.8; *SD* = 3.4) and the others (*M* = 10.8; *SD* = 6.6), with a small between-group difference (*β* = −0.651 (95% CI: −4.518, 3.215), *p* = 0.7).

## Discussion

This study provides preliminary evidence for positive associations between daytime sleepiness and emotional/behavioral disturbances, independent of nighttime sleep-disordered breathing, sleep adequacy, and BMI. Regarding the secondary aim, age but not BMI moderated this association: daytime sleepiness was more strongly associated with emotional/behavioral problems in children than in adolescents or young adults. The last finding is not surprising because in children without PWS, daytime sleepiness often manifests as emotional/behavioral problems [26]. Indeed, narcolepsy in children (but not adults) may first manifest itself not by excessive daytime sleepiness, but through apparent emotional/behavioral disturbances such as hyperactivity, emotional dysregulation, and aggression [27].

After adjusting for sleep adequacy, sleep-related breathing difficulties, and BMI, statistically significant associations were found between daytime sleepiness and all subscales of the DBC except communication disturbance. The 13-item communication disturbance subscale includes items such as confusing pronouns, being over-affectionate, overly interested in mechanical things, preferring adult company, echolalia, verbal repetition, whispering, standing too close, and talking to self. These behaviors were not associated with excessive daytime sleepiness in the adjusted model. By contrast, the 29-item autism subscale (while overlapping the communication disturbance subscale by 4 items) also includes items regarding tantrums, impatience, restlessness, poor sense of danger, repetitive movements, screaming, throwing things, and distress over small changes in routine. As such, the communication disturbance domain does not equate to the autism screening algorithm where the latter behaviors were more likely to occur in children with excessive daytime sleepiness.

The individuals with PWS in the present study showed a high prevalence of and a diverse range of emotional/behavioral disturbances, and this is consistent with previous studies [8, 9]. Many of these children displayed high levels of disruptive and self-absorbed behaviors and experience anxiety and depression. Pediatricians often encounter difficulties in supporting children with challenging emotional/behavioral problems and their families, and clarification of mechanisms applicable to PWS would enhance their capacity to implement comprehensive patient-centered care [28].

These mechanisms are complex and not well understood. In non-PWS populations, biopsychosocial models have been developed to explain associations between nighttime sleep disturbances and emotional/behavioral disturbances. These models highlight the mechanisms by which impaired psychological (e.g., rumination, loneliness) and cognitive (e.g., memory, attention) functioning can arise from sleep disorders, and other effects of biological factors and comorbidities [29–31]. Moreover, these models also identify multiple points along the causal pathways at which interventions may be implemented to modify downstream expression of emotional, cognitive, and emotional/behavioral disturbances.

For PWS, complex models describing the relationships between sleep and emotional/behavioral problems would need to take into account the roles of genetic influences, the hypothalamic–pituitary–adrenal pathway, cognitive functioning, and social functioning difficulties [32]. One possible mechanism may involve neuro-circuitries in the hypothalamus or the ventral tegmental area (VTA). The mechanisms involved in narcolepsy and attention deficit hyperactivity disorder (ADHD) may provide some parallels to the possible mechanisms in PWS. Narcolepsy with cataplexy (sudden loss of muscle tone), known as narcolepsy type 1 (NT1), is caused by low levels of hypocretin-1 [33], which is one of the key neurotransmitters sustaining wakefulness and alertness. The levels of hypocretin in the cerebral spinal fluid of individuals with PWS has been found intermediate between those of individuals with narcolepsy and individuals with idiopathic hypersomnia [34]. Hypocretin-producing neurons also innervate neurons in the VTA that produce dopamine for the neocortex. ADHD is caused by dopamine dysregulation of the meso-cortical pathway, leading to cortical hypoarousal, inattention, executive dysfunction, and poor self-control, which can in turn cause emotional dysregulation, irritability, and emotional/behavioral disturbances [35].

Pharmacological treatment of emotional/behavioral disturbances in PWS is problematic because there is weak evidence for the efficacy of any psychotropric medication used singly for PWS, and combining medications increases the risk of adverse effects [36]. There is emerging evidence that pitolisant, a histamine 3 receptor inverse agonist, may be an effective alternative mechanism to exploit for both sleepiness and emotional/behavioral disturbances. Evidence from case studies of 4 children and adolescents with PWS indicates that pitolisant can reduce daytime sleepiness while improving attention and cognition, and, reducing tantrums and aggressive behavior [36, 37]. Pitolisant can have adverse effects, including cardiac side effects, which may need to be considered and monitored. Our observational study suggests that intervention studies are indicated [36].

More generally, assessment of the adequacy of sleep duration and screening for common sleep disorders, including OSA [5, 28], continue to be important parts of long-term management of PWS. Improvements in sleep habits, particularly regular bedtimes, have been demonstrated to yield positive outcomes for sleep duration, nighttime awakenings, and daytime emotional/behavioral problems in typically developing children [38, 39].

### Limitations

The distributions of features such as genetic subtype and BMI in our sample reflected observations in larger studies [40, 41]. Nevertheless, our analysis was limited by sample size which necessitated a parsimonious approach when choosing covariates and was insufficient to examine other factors (such as genetic subtypes and other comorbidities) that might also influence emotional/behavioral disturbances. Our assessment of sleep-related breathing difficulties depended on parent report in the PSQ; the methodology did not permit calculation of an apnea–hypopnea index. More objective information collected from polysomnography would be desirable but was not practical across this age range and for a national sample for which polysomnography is not routinely used. There was no evidence from this study that growth hormone influenced daytime sleepiness or breathing difficulties, but very few were not taking growth hormone (*n* = 13), so the study had little power to detect an effect. The effects of stimulants on behavior could not be examined, as the number of children were being treated for narcolepsy (*n* = 3) was too small; however, this is likely to weaken the observed association between daytime sleepiness and emotional/behavioral disturbances. We acknowledge suggestions that there could be relationships between the main genetic subgroups and specific emotional/behavioral disturbances, but at present correlations between genotypes and phenotypes are inconsistent, most likely because the PWS critical region encompasses five protein-coding genes and more than 80 RNA genes [42]. Given the cross-sectional design of this study, causal relationships cannot be examined, and therefore, extrapolated causal inferences need to be tested in further longitudinal and experimental studies. An experimental study with an intervention to target daytime sleepiness could potentially test the causal roles of daytime sleepiness on emotional and emotional/behavioral disturbances.

## Conclusions

The study shows that daytime sleepiness appears to be associated with emotional/behavioral disturbances in children and young people with PWS, independently of nighttime sleep adequacy, symptoms of sleep-disordered breathing, and BMI. Our cross-sectional study design precludes causal inferences, but our findings nevertheless provide preliminary evidence to support further investigations to support proceeding to a “proof of concept” study, testing whether pharmacological treatment targeting daytime sleepiness may be effective for managing daytime emotional/behavioral disturbances.

## Data Availability

The data that support the findings of this study are available from the corresponding author upon reasonable request and ethics approval.

## Abbreviations

BMI: Body mass index
CSA: Central sleep apnea
DBC-P: Developmental Behavior Checklist—parent report
ESS-CHAD: Epworth Sleepiness Scale for Children and Adolescents
OSA: Obstructive sleep apnea
PSQ: Pediatric Sleep Questionnaire
PWS: Prader-Willi syndrome
REDCap: Research Electronic Data Capture platform
